# Supplementary material for: A Conserved Cysteine Residue in Coxsackievirus B3 Protein 3A with Implication for Elevated Virulence
Source: Viruses. 2022 Apr 7;14(4):769. doi: 10.3390/v14040769 (PMC9029043; doi:10.3390/v14040769)
Supplement: Supplementary file 1 [file viruses-14-00769-s001.zip › viruses-1659845-supplementary.pdf]

viruses

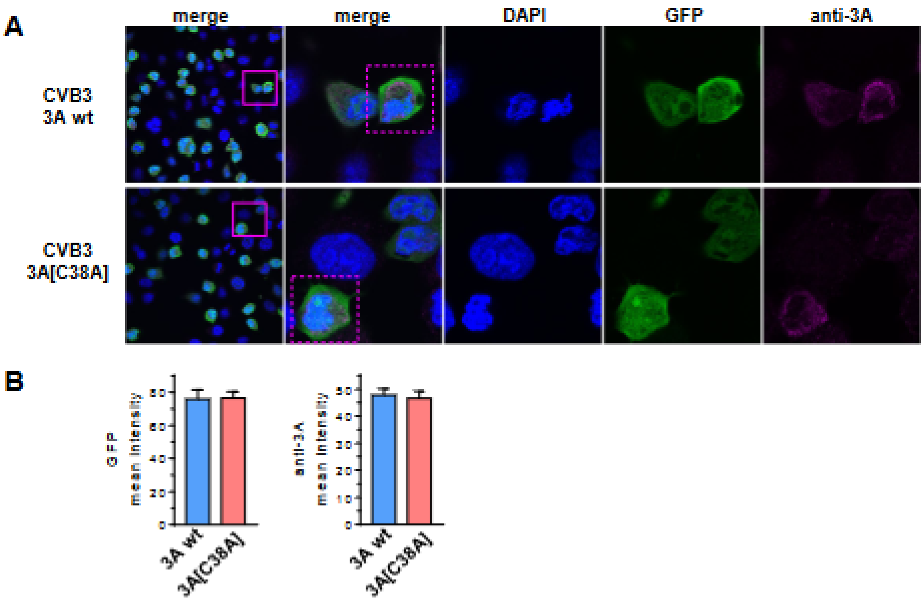

Figure S1. (for Figure 2): Quantitative comparison of intensity distributions and autocorrelation-based image correlation spectroscopy. **(A)** For the analysis of anti-3A immunofluorescence distribution in cells infected with GFP-CVB3 encoding protein 3A wt or 3A[C38A], cells with similar intensities (dashed square) of the GFP fluorescence and anti-3A immunofluorescence were selected. **(B)** The bar charts depict the mean intensities of GFP and anti-3A signals of selected cells used for the quantitative comparison of intensity distributions and autocorrelation-based image correlation spectroscopy (n=15).
